# T-tube Duodenostomy for the Difficult Duodenum

**DOI:** 10.7759/cureus.32965

**Published:** 2022-12-26

**Authors:** Adina McNair, Patrick D Melmer, Aaron D Pinnola

**Affiliations:** 1 General Surgery, Grand Strand Medical Center, Myrtle Beach, USA; 2 Acute Care Surgery, Virginia Commonwealth University, Richmond, USA; 3 Hepatopancreaticobiliary Surgery, Grand Strand Medical Center, Myrtle Beach, USA

**Keywords:** tube duodenostomy, duodenostomy tube, duodenal stump, acute care surgery, foregut surgery, duodenal perforation

## Abstract

Tube duodenostomy has been described as a useful technique in the management of difficult duodenum arising from a variety of pathologies. In addition, the use of a t-tube for the duodenostomy presents a resourceful option in the event of Malecot or other such catheter unavailability. The aim of our study is to describe the technique and outcomes associated with this approach.

During a six-month period in 2020, t-tube duodenostomies were performed in three patients for duodenal stump perforation: the first case involved a patient with Roux-en-Y esophagojejunostomy anatomy; the second involved duodenal stump closure security following Billroth II gastrectomy for peptic ulcer disease; and the third involved decompression following primary closure of duodenal perforation. All duodenostomies were performed with a t-tube that was trimmed with the back wall divided and then secured via the Witzel approach.

The t-tube duodenostomies were performed during the index operations of all patients. No patient required additional operations. There was no mortality. All patients were closely monitored postoperatively with duodenostomies kept in place for six weeks. One patient developed a small leak after a trial of tube clamping, which was managed with continued tube drainage and antibiotics prior to definitive removal. The mean length of stay was 20.3 days with two patients being discharged to rehab.

T-tube duodenostomy is a simple technique that helps avoid the blowout of the vulnerable duodenal stump in situations of biliopancreatic limb pathology, ulcerative disease, or injury.

## Introduction

The management of challenging duodenal pathology has been a topic of discussion in the surgical literature. There have been a variety of approaches proposed to best address the closure of the difficult duodenal stump [1]. However, primary closure of the duodenal stump can be arduous in many cases due to the presenting anatomic pathology. In such cases, the use of tube duodenostomy has been described as a useful technique in the management of a variety of duodenal pathologies [2-7]. Many cases have reported the use of a Malecot catheter as a duodenostomy tube [3]. In this case series, we describe the use of a t-tube. The t-tube duodenostomy presents a resourceful alternative in the event of Malecot or other such catheter unavailability. The aim of our study is to describe the technique and outcomes associated with this approach.

This article was previously presented as a meeting abstract at the Southeastern Surgical Conference 2021 Annual Meeting on August 23, 2021.

## Case presentation

**Figure 1:**
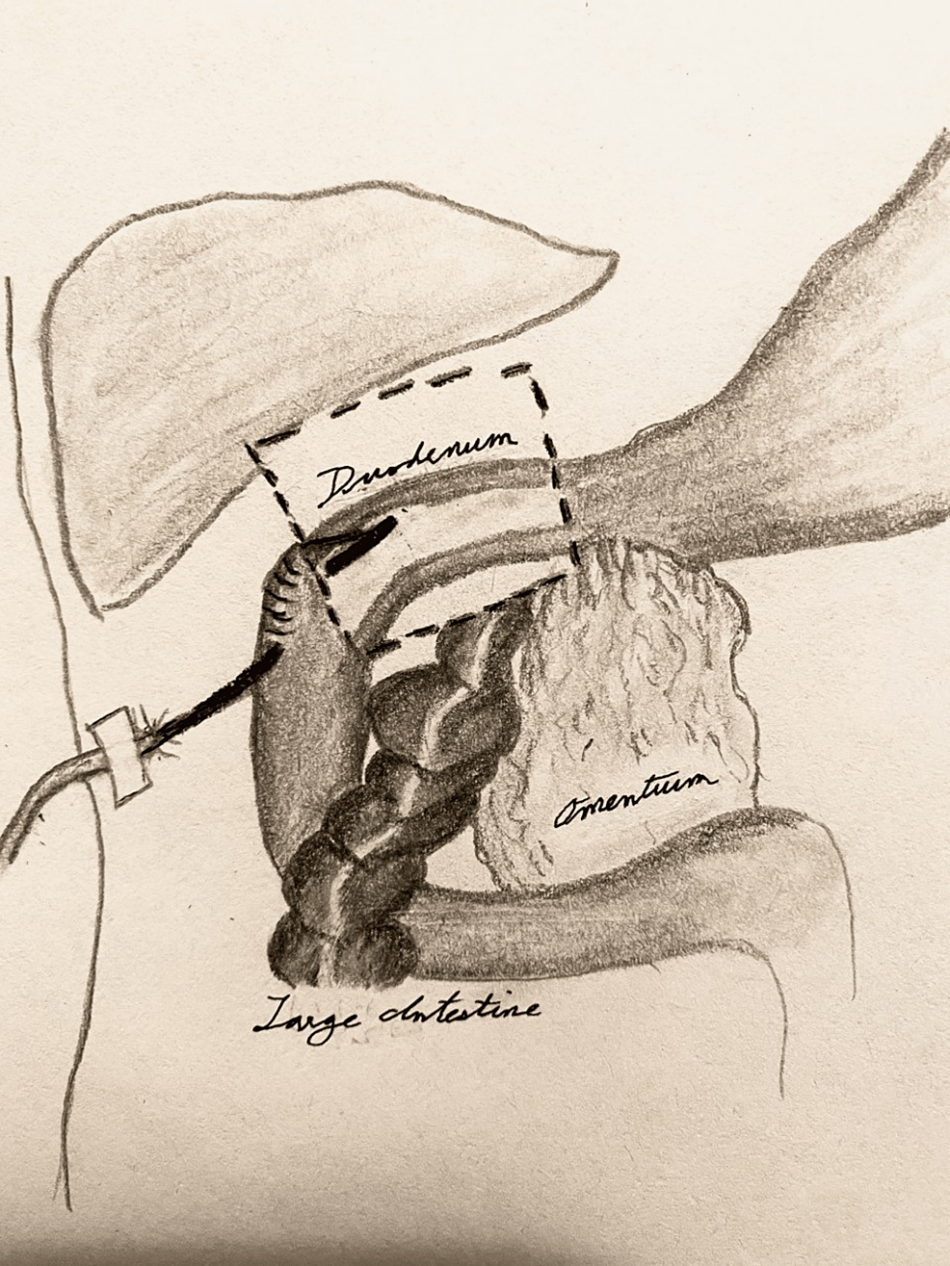
Illustration of t-tube duodenostomy secured via the Witzel approach. Illustration by Emma Rousakis, MD.

During a six-month period in 2020, t-tube duodenostomies were performed in three patients for duodenal stump drainage: the first case involved a duodenal stump perforation in a patient status post remote Roux-en-Y esophagojejunostomy; the second involved a duodenal stump closure in conjunction with Billroth II (BII) gastrectomy for peptic ulcer disease; and the third involved decompression following primary closure of duodenal ulcer perforation. All patients underwent placement of t-tube duodenostomy plus external drainage with the placement of a round, closed-suction drain near the duodenum. Each of the three patients also had placement of nasogastric tube and distal feeding jejunostomy. All duodenostomies were performed with a 14 or 16 French t-tube that was trimmed with the back wall divided and then secured via the Witzel approach (Figure 1). All duodenostomy tubes were removed successfully at six weeks postoperatively after first undergoing a trial of clamping.

Case 1

A 77-year-old male with a history of hiatal hernia surgery in 2018 complicated by gastric necrosis requiring total gastrectomy with Roux-en-Y esophagojejunostomy presented with abdominal pain and distension. CT imaging demonstrated distention of the biliopancreatic limb of Roux-en-Y concerning for stasis or obstruction. He had a trial of nonoperative management with nasogastric tube decompression, fluid resuscitation, and antibiotics. The patient subsequently developed hypotension and worsening abdominal distension that day and was taken to the operating room for exploratory laparotomy. He was found to have small bowel obstruction as well as areas of full-thickness necrosis of the first and second portions of the duodenum with a perforation in one of the areas of necrosis. He underwent primary repair of the duodenal perforation with a serosal patch. Due to the severity of tissue necrosis and surrounding tissue/bowel friability, the patient underwent placement of decompressive t-tube duodenostomy placed distally in the healthy portion of the duodenum, followed by closed-suction drains placed anterior and posterior to the duodenal stump. A nasogastric tube was placed in the gastric pouch. Following the lysis of adhesions surrounding the jejunojejunostomy that was the source of obstruction, a jejunostomy tube was placed distal to the jejunojejunal anastomosis. The abdomen was closed using retention sutures due to a history of a large ventral hernia.

Case 2

An 80-year-old female presented with dizziness, syncope, and a large volume of melenic stool. The patient underwent esophagogastroduodenoscopy, which demonstrated a large, bleeding posterior duodenal ulcer, which was not amenable to endoscopic intervention. She underwent coil embolization of the gastroduodenal artery by interventional radiology. Later, she had persistent large-volume melenic stools and hemodynamic instability, which prompted a surgical intervention. The patient was taken for an exploratory laparotomy and underwent an antrectomy with partial resection of the duodenum and BII reconstruction. The duodenum was primarily approximated in two layers secondary to a high degree of inflammation, tissue friability, and large defect size. Therefore, a duodenostomy tube, distal feeding jejunostomy, and anterior and posterior drains were placed.

Case 3

A 73-year-old female presented with abdominal pain for which cross-sectional imaging was obtained. This demonstrated free air and concern for perforated peptic ulcers. The patient was taken for exploratory laparotomy, where she was found to have a 2 cm giant perforated ulcer on the superior aspect of the first portion of the duodenum. The most posterior aspect of the ulcer was immediately adjacent to the common bile duct, making primary repair not feasible. The majority of the ulcer was primarily approximated with the posterior aspect covered with an omental patch. Given the risk of stump leak, a duodenostomy tube was placed along with a closed-suction drain near the duodenum and distal feeding jejunostomy. The patient then underwent a pyloric exclusion with gastrojejunostomy.

T-tube duodenostomies were performed during the index operations of all patients due to underlying anatomic pathology that did not allow for primary definitive closure. No patient required additional operations. There was no mortality. All patients were closely monitored postoperatively, with duodenostomies kept in place for six weeks. One patient developed a small duodenal stump leak after a trial of tube clamping, which resolved with continued tube drainage and antibiotics, and the tube was still removed at six weeks postoperatively, as planned. The mean length of stay was 20.3 days, with two patients being discharged to rehabilitation facilities (due to associated comorbidities and deconditioning during the hospital stay).

## Discussion

Duodenal leaks are a feared complication that can arise from a variety of etiologies following either resection, injury, or repair of perforations. The overall mortality rate of duodenal stump leak has been cited as between 15% and 75% [8-12]. Emergency operations in the setting of acute tissue inflammation and friability increase the risk of leaks. Po Chu Patricia et al. were one of the first groups to look at duodenal leak rates and complications for both elective and emergency gastrectomies [8]. They reported a total stump leak rate of 7.7% and showed a significant increase in the duodenal stump leakage rate of 21% compared to the 3.4% in the elective group [8]. It has been shown that the management of the duodenum in these high-risk scenarios is feasible and safe by using a duodenostomy tube for decompression and drainage [2-7].

Different closure techniques in an attempt to decrease the risk of leak after gastric resection have been described [1]. However, in settings of contamination, friable tissue, and medical comorbidity, they are unable to safely perform definitive closure of the duodenal stump. For this reason, we believe the placement of a duodenostomy tube for drainage in these patients is the best option to decompress the duodenum and decrease the need for re-operation due to stump leaks. A cohort study of 678 patients identified four independent risk factors for duodenal stump leak: lower pre-operative hemoglobin, contamination, the existence of duodenal ulcer, and the use of a duodenostomy tube [8]. However, when they divided the patients into subgroups of emergency vs. elective gastrectomy, it was reported that the use of a duodenostomy tube did not have a significant association with duodenal stump leaks in the emergency surgery group. This suggests an area for further research and investigation to identify risk factors where a duodenostomy tube may be advantageous, such as perforated ulcers, severe inflammation, or acute hemorrhage. In this case series, we describe the technique of decompressive tube duodenostomy via a t-tube in lieu of the traditionally described Malecot drain. While this technique was born out of necessity secondary to Malecot drain unavailability, we found this technique to achieve excellent results. To our knowledge, this is the first published description of decompressive duodenostomy via a t-tube technique. Further propagation of this strategy and subsequently published data are needed to definitively state superiority to previously described techniques.

## Conclusions

T-tube duodenostomy is a simple and safe technique that helps avoid the blowout of the vulnerable duodenum in situations of biliopancreatic limb pathology, ulcerative disease, or injury. Although some studies may still argue against the use of duodenostomy tubes, many others report the safety and efficacy of this technique. We add to these data by demonstrating a modification to the usual tube duodenostomy technique utilizing a t-tube secured via the Witzel approach. The t-tube duodenostomies were performed at the index operation of all three patients presented. There was no mortality and no additional operations were required. All patients were able to have the duodenostomy tubes removed at six weeks postoperatively and all fistula tracts closed without complication following removal. Given this success, in emergency settings where definitive primary closure of the stump is not possible, we propose the consideration of T-tube duodenostomy as an effective method for duodenal drainage and decompression as a safe alternative to larger resections or previously described closures.
